# Comorbid alcohol and cannabis use disorders increase mortality in patients with eating disorders

**DOI:** 10.1192/j.eurpsy.2022.345

**Published:** 2022-09-01

**Authors:** L. Stryhn, A. Mejldal, M. Guala, R. Støving, E. Stenager, L. Skøt, A. Mellentin

**Affiliations:** 1 Unit for Psychiatric Research, Department of Clinical Research, University Of Southern Denmark, Odense C, Denmark; 2 Center for Eating Disorders, Odense University Hospital, Odense C, Denmark; 3 Research Unit for Medical Endocrinology, Institute Of Clinical Research, University Of South Denmark, Odense, Denmark; 4 Unit for Psychiatric Research, Institute Of Regional Health Services Research, University Of Southern Denmark, Aabenraa, Denmark; 5 Brain Research-Inter-Disciplinary Guided Excellence (BRIDGE), Department of Clinical Research, University Of Southern Denmark, Odense, Denmark

**Keywords:** Eating Disorders, cannabis use disorder, Alcohol use disorder, mortality

## Abstract

**Introduction:**

Alcohol and cannabis use disorders are the most frequent comorbid substance use disorders (SUDs) among patients with eating disorders (EDs). EDs and SUDs involving alcohol and cannabis are independently associated with excess mortality.

**Objectives:**

To investigate the impact of comorbid alcohol use disorder (AUD) and cannabis use disorder (CUD) on mortality in anorexia nervosa (AN), bulimia nervosa (BN), and unspecified eating disorder (USED) compared with matched control subjects.

**Methods:**

This retrospective cohort study was conducted using Danish nationwide registers. The risk of mortality among ED patients with/without AUD and/or CUD was compared to matched control subjects with/without AUD and/or CUD using hazard ratios (HRs).

**Results:**

Of the 20,759 included ED patients, 4.7% and 4.3% had AUD and CUD, respectively. The corresponding figures for the 83,036 control subjects were 1.0% (AUD) and 1.3% (CUD). ED patients without SUDs exhibited an increased risk of mortality compared to control subjects without SUDs (adjusted HR 2.9, *P*<.001). Mortality risk was higher among ED patients with AUD (adjusted HR 11.8, *P*<.001) or CUD (adjusted HR 4.6, *P*<.001) compared to control subjects without AUD/CUD. In addition, patients with AN, BN, and USED, who had comorbid AUD and/or CUD, exhibited an elevated risk of mortality compared to control subjects without AUD/CUD (AN: adjusted HR 11.3, *P*<.001; BN: adjusted HR 5.9, *P*<.001; USED: adjusted HR 10.9, *P*<.001).

**Conclusions:**

Comorbid AUD and/or CUD increase mortality risk in patients with EDs. In order to reduce mortality in ED patients, prevention and treatment of AUD and CUD is important.

**Disclosure:**

No significant relationships.

